# Allen Oldfather Whipple (1881-1963): A Pioneer of General Surgery

**DOI:** 10.7759/cureus.88895

**Published:** 2025-07-28

**Authors:** Ledio Gjunkshi, Lorela Gjunkshi, Nadiya A Persaud, Sanjiv F Gray

**Affiliations:** 1 Research, Orlando College of Osteopathic Medicine, Winter Garden, USA; 2 Research, Saba University School of Medicine, The Bottom, NLD; 3 Surgery, Orlando College of Osteopathic Medicine, Winter Garden, USA; 4 Surgery, Lakeland Regional Health, Lakeland, USA

**Keywords:** allen oldfather whipple, pancreatic insulinoma, surgical history, whipple procedure, whipple triad

## Abstract

Dr. Allen Oldfather Whipple (1881-1963) was a pioneer in general surgery whose innovative techniques transformed the management of pancreatic insulinomas and continue to shape modern surgical practices today. Born in Iran to missionary parents, Dr. Whipple later moved to the United States in pursuit of greater educational opportunities. He spent the majority of his medical career in New York, leaving a lasting impact on the world through his leadership and surgical expertise. Between the 1930s and 1940s, he developed the Whipple procedure (pancreaticoduodenectomy), which has since evolved through advances in surgical technique, instrumentation, and perioperative care to remain the gold standard for treating pancreatic head malignancies. In 1935, he also introduced the Whipple triad, which remains central in the diagnosis of insulinoma. Dr. Whipple served as president of several leading surgical organizations, including the American Surgical Association, where he advocated for surgical education reform and promoted standardization of operative techniques. He also authored three influential books: “Surgical Diagnosis,” “Surgery of the Pancreas,” and “Techniques of Abdominal Operations,” which became foundational texts in surgical training. This historical vignette showcases the life and legacy of Dr. Allen Oldfather Whipple, highlighting his profound impact not only on his local hospital but also on the global medical community.

## Introduction and background

Early life

**Figure 1:**
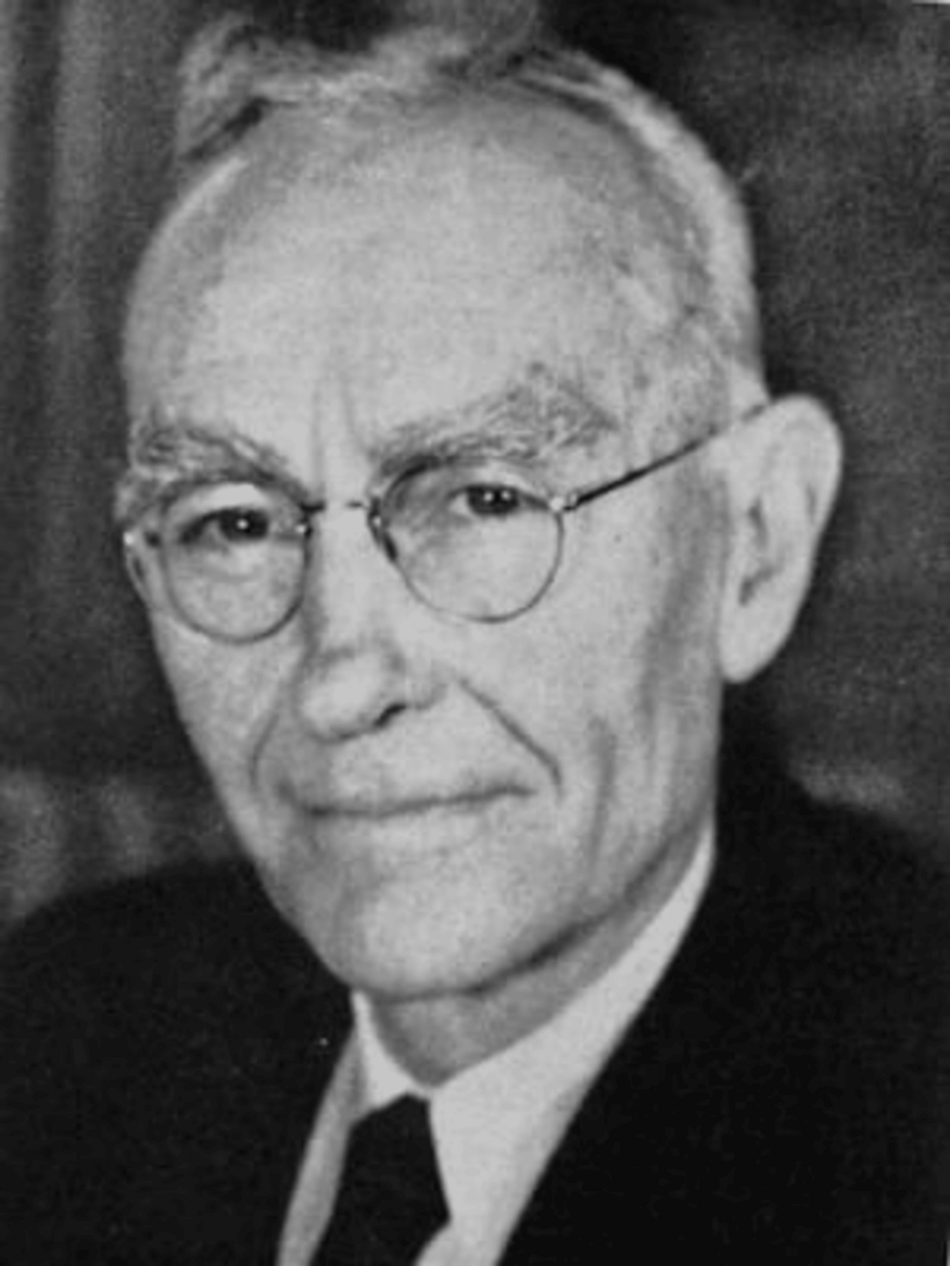
Portrait of Dr. Allen Oldfather Whipple Portrait from Mike Cadogan, LITFL: Allen Oldfather Whipple (Nov 3, 2020). Licensed under CC BY‑NC‑SA 4.0. [7]

Allen Oldfather Whipple (Figure 1) was born on September 2, 1881, in Urmia, Persia (modern-day Iran), a city that at the time served as a significant center for missionary and medical outreach in the region, particularly through the work of American Presbyterian missions [1-2]. His parents, William Levi Whipple and Mary Louise Allen, were American Presbyterian missionaries stationed there [2-3]. Although the couple had three children before him who sadly passed away in infancy, Allen was the eldest of their five surviving children [4]. Growing up in a missionary household instilled in him values of service, discipline, and compassion, all of which would later shape his approach to medicine and leadership. He spent his early years in Iran before immigrating to the United States with his family in 1896 at the age of 15 [5-6].

Undergraduate and medical education 

In 1904, Whipple earned his Bachelor of Science degree from Princeton University in New Jersey [2,7]. At Princeton, he studied biology and chemistry, which laid the academic foundation for his later interest in physiology and surgical science. He was drawn to medicine through his personal experience with typhoid fever and surviving a cholera epidemic. His interest in surgery was sparked after his sister underwent surgery to treat a bowel obstruction [6]. Whipple went on to complete his medical education in New York at the Columbia University College of Physicians and Surgeons, where he earned his medical degree in 1908 [2,7].

Medical training

Dr. Whipple trained at Roosevelt Hospital in New York, where he completed a two-year internship. His training emphasized abdominal and gastrointestinal surgery, which would later inform his groundbreaking work on the pancreas. Upon completing his internship, he assumed the role of associate professor and later became the chairman of the Department of Surgery. In 1921, Dr. Whipple joined the surgical staff at Columbia-Presbyterian Hospital in New York, where he held the position of Director of the Surgical Service [2,5,7]. During his tenure, he expanded surgical residency training programs, emphasized scientific rigor in operative technique, and mentored future leaders in academic surgery. He was also the first to hold the title of Valentine Mott Professor of Surgery, a position he obtained in 1931. The Valentine Mott Professorship, named after a pioneering 19th-century American vascular surgeon, is one of the most prestigious academic surgical appointments at Columbia, recognizing exceptional leadership and contributions to surgical education and research [2,8-9].

Pancreatic insulinomas are rare, insulin-secreting neuroendocrine tumors that can lead to recurrent and severe episodes of hypoglycemia [2-5]. Prior to Dr. Whipple’s surgical innovations, diagnosing and treating these tumors posed significant clinical challenges. His work provided a reliable diagnostic framework, the Whipple triad, and introduced an innovative surgical approach, markedly improving patient prognosis.

## Review

Surgical innovations

In 1935, while serving as Chief of Surgery at Columbia-Presbyterian Hospital, Dr. Whipple was performing a gastrectomy before an audience, planning to remove part of the stomach from a patient diagnosed with gastric cancer [4]. During the procedure, he discovered that the patient instead had pancreatic cancer. Forced to improvise, he extended the resection to include not only the stomach but also the pancreatic head, duodenum, jejunum, and common bile duct, marking a significant departure from prior approaches that had only partially removed affected tissues. The operation was a success and was later named the Whipple procedure, though its anatomical term is pancreaticoduodenectomy [2-5,7]. Initial reports noted a high perioperative mortality rate (~25-30%), but refinements in surgical technique, anesthesia, and postoperative care have since improved outcomes, with current five-year survival rates reaching up to 20-25% for select cases of pancreatic cancer [7].

Credit is also given to Dr. Walther Kausch, a German surgeon who performed a similar but limited pancreaticoduodenectomy in 1909. Some references use the term Kausch-Whipple operation to acknowledge both contributions; however, this name is not widely adopted [2,8].

Diagnostic contributions

**Figure 2:**
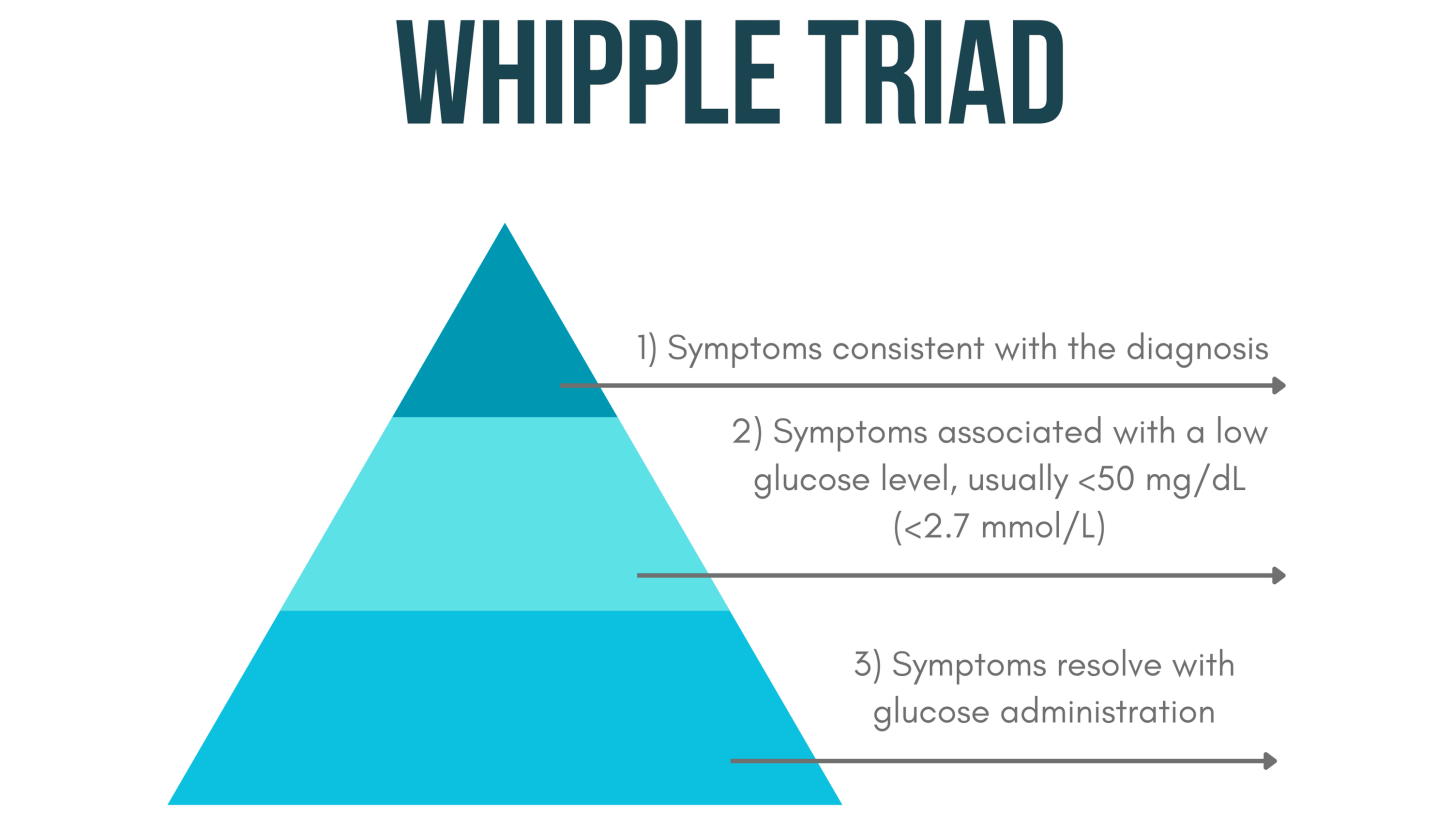
Whipple triad Infographic designed by Nadiya A Persaud on Canva. Software: Canva Pro (Canva Inc., Perth, Australia)

Following the success of his complete pancreaticoduodenectomy, Dr. Whipple sought to better understand how pancreatic tumors such as insulinomas could be misdiagnosed. In 1938, he introduced a diagnostic framework known as the Whipple triad to aid in the identification of pancreatic insulinomas [7]. The Whipple triad includes (1) hypoglycemia with clinical signs and symptoms, (2) blood glucose level under 50 mg/dL, and (3) relief of symptoms following glucose administration (Figure 2) [5,7-9].

The triad remains clinically relevant and is still referenced in contemporary endocrinology, particularly in the evaluation of hypoglycemia. However, it is now often supplemented with additional biochemical tests and imaging modalities for diagnosis [9].

Legacy and later life

Dr. Whipple married Mary Neales, with whom he had three children: Mary Allen Whipple, Allen Oldfather Whipple Jr., and William Neales Whipple [6]. Tragically, William died in a car accident in 1933 at the age of 16 despite the efforts of Columbia-Presbyterian’s surgical team [6].

Dr. Whipple’s contributions to surgery are far-reaching. The Whipple procedure remains one of the most complex yet effective surgical interventions for pancreatic cancer [4]. Beyond this, he served as president of numerous surgical organizations, including the New York Surgical Society (1934), the Society of Clinical Surgery (1936), and the American Surgical Association (1940) [7].

After retiring in 1946, Dr. Whipple continued his work as Clinical Director at Memorial Sloan Kettering Cancer Center for four years [2]. In this role, he helped modernize surgical oncology protocols and emphasized the integration of basic science with surgical practice, contributing to the early development of multidisciplinary cancer care. He later served as a visiting professor at the American University of Beirut and held the title of Valentine Mott Professor Emeritus at Columbia University [2].

During retirement, he authored three books: The Story of Wound Healing and Wound Repair, The Evolution of Surgery in the United States, and The Role of the Nestorians and Muslims in the History of Medicine [2]. The latter text is especially notable for its historical exploration of medical knowledge exchange across cultures, emphasizing the contributions of Middle Eastern scholars and bridging gaps in Western medical historiography.

Dr. Allen Oldfather Whipple passed away on April 16, 1963, in Princeton, New Jersey [2,4]. His legacy endures through the global adoption of the Whipple procedure, now considered the gold standard for resectable pancreatic-head tumors [10]. He also played a key role in founding the American Board of Surgery, helping to establish formal credentialing for general surgeons and create a lasting contribution to surgical standards and education [11].

## Conclusions

Dr. Allen Oldfather Whipple was an esteemed and respected surgeon who spent most of his career at Columbia-Presbyterian Hospital in New York. He revolutionized the surgical treatment of pancreatic insulinomas by developing and refining the Whipple procedure and introduced the Whipple triad in 1938 to aid in the diagnosis of insulinomas. While the triad remains clinically relevant, it is now typically used in conjunction with modern biochemical testing and imaging protocols.

The Whipple procedure has undergone significant advancements since its original development, including improvements in surgical technique, anesthesia, and perioperative care, contributing to reduced mortality and enhanced patient outcomes. It is now widely regarded as the gold standard for resectable tumors of the pancreatic head and is routinely referenced in surgical textbooks and international oncology guidelines.

Beyond his technical innovations, Dr. Whipple was a dedicated educator and leader who helped shape the field of modern surgery through his mentorship, his presidency in key surgical societies, and his efforts to advance surgical education and credentialing. His enduring influence continues to inspire generations of surgeons and cements his legacy as a pioneer in both surgical practice and medical education.
